# Comparative effectiveness of seven interventions for chronic hepatitis D: a systematic review and network meta-analysis of randomized controlled trials

**DOI:** 10.1186/s12879-023-08718-7

**Published:** 2023-10-25

**Authors:** Yangdan Rong, Xuegui Ju, Peng Sun, Yali Wang

**Affiliations:** 1 Department of Infectious Diseases, The First Affiliated Hospital of Chengdu Medical College, Chengdu, Sichuan 610500 China; 2 Department of General Practice, The First Affiliated Hospital of Chengdu Medical College, Chengdu, Sichuan 610500 China; 3 Department of Cardiothoracic Surgery, The First Affiliated Hospital of Chengdu Medical College, Chengdu, Sichuan 610500 China

**Keywords:** Chronic hepatitis D, Interferon, Nucleoside analogs, Bulevirtide, Randomized controlled trial, Network meta-analysis

## Abstract

**Objective:**

To compare the effectiveness of seven major interventions [Bulevirtide (BLV), Interferon (IFN), Nucleoside analogs (NAs), BLV + IFN, BLV + NAs, IFN + NAs, and Placebo] to treat chronic hepatitis D.

**Methods:**

We followed PRISMA-NMA guidelines, searched databases (Cochrane Library, PubMed, EMBASE, and Web Of Science) for eligible randomized controlled trials (RCTs), and applied STATA17.0 software to execute the meta-analysis.

**Results:**

We included 14 randomized controlled trials (814 patients) comparing seven different interventions. The results of the network meta-analysis showed that: ① Sustained virological response (after 24 weeks of follow-up): Four intervention groups (BLV + IFN, IFN alone, IFN + NAs, and NAs alone) were effective (relative risk (RR) = 13.30, 95% confidence interval (Cl) [1.68,105.32], RR = 12.13, 95% Cl [1.46,101.04], RR = 5.05, 95% Cl [1.68,15.19], RR = 5.03, 95% Cl [1.66,15.20]), with no statistically significant differences between the four groups. The top three in probability rankings were: BLV + NAs, BLV + IFN, and BLV alone (surface under the cumulative ranking curve (SUCRA) = 86.8%, 80.3%, and 48.4%; ② Sustained biochemical response (after 24 weeks of follow-up): BLV + IFN and IFN were superior to BLV (RR = 14.71, 95% Cl [1.14,189.07], RR = 16.67, 95% Cl [1.39,199.52]). The top three were BLV alone, BLV + NAs, and BLV + IFN (SUCRA = 86.9%,81.2%, and 64.3%). ③ Histological response: NAs were superior to BLV (RR = 2.08, 95% Cl [1.10,3.93]), whereas the difference between other treatment regimens was not statistically significant, and the top three in the probability ranking were BLV alone, BLV + NAs, and BLV + IFN (SUCRA = 75.6%, 75.6%, and 61.8%).

**Conclusions:**

IFN, IFN + BLV, and IFN + NAs were effective in clearing HDV RNA and normalizing alanine aminotransferase levels; however, IFN and IFN + NAs had a high rate of viral relapse at 24 weeks post-treatment follow-up. There was no additional benefit of adding NAs to IFN therapy for chronic hepatitis D; however, the combination of IFN + BLV significantly improved short-term HDV RNA clearance, which showed strong synergistic effects. The seven regimens included in the study did not contribute significantly to liver histological improvement. Therefore, the IFN + BLV combination has the most potential as a treatment option to improve the long-term prognosis or even cure chronic hepatitis D.

**Trial registration:**

This systematic evaluation and meta-analysis was registered with PROSPERO under the registration number: CRD42022314544.)

**Supplementary Information:**

The online version contains supplementary material available at 10.1186/s12879-023-08718-7.

## Background

Hepatitis D virus (or δ virus) (HDV) is a defective small single-stranded circular RNA virus that requires the co-function of hepatitis B virus (HBV) for viral assembly and transmission [1]. Among HBV-infected individuals, superinfection with hepatitis D virus leads to disease progression and cirrhosis in approximately 80% of patients. Cirrhosis develops earlier in patients with HDV infection than in those with chronic HBV infection only [2, 3]. A meta-analysis of the prevalence of hepatitis D estimated that approximately 587 million people worldwide are infected with HBV, of whom 62–72 million are co-infected with HDV [4].

At present, there is no HDV-specific drug approved by the Food and Drug Administration (FDA); however, standard PEGylated interferon (PEG-IFNα) therapy has been widely used as an anti-HDV treatment for the past several decades. In vitro model studies showed that replication of HDV was barely affected by interferon [5, 6]. The results observed in the clinic are significantly different from the in vitro model studies, where the response to interferon in patients with chronic hepatitis D is usually characterized by a decrease in HDV viral load and alanine aminotransferase (ALT) levels, suggesting a direct antiviral effect of interferon on HDV [7–9]. A 48-week course of PEG-IFNα treatment (subcutaneous injection, weekly) suppressed HDV replication in 20–30% of patients, but had significant side effects. Combination with adefovir for 48 weeks [10] or with tenofovir disoproxil fumarate (TDF) for 96 weeks did not significantly improve HDV RNA clearance [11]. Significantly, a late virological relapse was observed in approximately 56% of virologically responsive patients followed up for 24 weeks after the end of treatment, further challenging the long-term effectiveness of this treatment [12].

The HDV life cycle has been studied in more detail in recent years, facilitating the development of specific antiviral drugs, and future options available for treatment are being innovated, including the entry inhibitor bulevirtide (Myrcludex-B, BLV); the nucleic acid polymer rep2139 CA, which inhibits HBV surface antigen secretion; an isopropylation inhibitor, Lonafarnib (LNF), which targets viral assembly; and a better-tolerated interferon, IFN-λ. Notably, BLV received conditional marketing authorization from the European Union (EU) in 2020 [13].

Bulevirtide, a myristoylated synthetic lipopeptide corresponding to the preS1 sequence of HBV surface antigen (HBsAg), has been shown to competitively inhibit HBV and HDV binding to hNTCP (human sodium taurocholate co-transporting polypeptide), thereby blocking the entry of HDV particles into hepatocytes [14–16]. In the MYR202 and MYR203 study, most patients treated with 10 mg BLV achieved at least 2 log HDV RNA decline or undetectable, the viral response rate of the BLV group was higher than that of the TDF group and the IFN group, and a significant synergistic effect was observed during the treatment [17–19]. This confirmed the good safety profile, sustained antiviral and ALT relapse efficacy of BLV. Most of the current systematic evaluations are direct comparisons of interferon therapy, and there is no comprehensive assessment of comparisons among polypharmacy regimens. Therefore, the present study aimed to apply network meta-analysis to comprehensively analyze virological, biochemical, and histological responses in patients with chronic hepatitis D treated with multiple interventions to provide more accurate and reliable evidence for clinical rational drug use.

## Methods

### Search strategy

The Cochrane Library, PubMed, EMBASE, and Web Of Science were searched with the search terms (“Hepatitis D” [Mesh] OR “Hepatitis D, Chronic” [Mesh]) AND (“Randomized Controlled Trials as Topic” [Mesh] OR “Randomized Controlled Trial” [Publication Type] OR (Clinical Trials, Randomized [Title/Abstract]) OR (Trials, Randomized Clinical [Title/Abstract]) OR (Controlled Clinical Trials, Randomized [Title/Abstract]) up to October 2022, to identify randomized clinical trials and search for bibliographical references of identified randomized trials, textbooks, and review articles relevant to this systematic review. Details of the search strategy were presented in Additional file 1.

### Inclusion criteria

The following criteria were required for inclusion: (1) The studies were randomized controlled trials; (2) The study population was patients with chronic hepatitis D (chronic hepatitis D was diagnosed when HDV-RNA levels and HDV antibodies were detected in serum at least 6 months before the study [20]), who were followed for 24 weeks after treatment with different regimens; (3) The sample size was at least 20 in a study with a follow-up period of at least 24 weeks. (4) Virological response, biochemical response, and histological response at the end of treatment (EOT) or 24 weeks of follow-up (FU24W) were recorded. (Virological response was defined as undetectable HDV RNA in serum at the end of treatment or 24 weeks of follow-up, and biochemical response was defined as was defined as the normalization of serum ALT (≤ 1xULN) at the end of treatment or 24 weeks of follow-up. Histological response was defined as a ≥ 2 decrease of the Knodell score or a ≥ 1 decrease of the Ishak score after treatment).

### Exclusion criteria

The study type was not clearly stated; there were problems with the trial design, such as inconsistent diagnostic criteria and evaluation indicators, unclear sample data submission, and the inability to extract valid outcome data; the study population was combined with HAV, HCV, HEV, and HIV infections and hepatocellular carcinoma, and included patients who were pregnant and lactating.

### Quality evaluation

Two authors independently evaluated the quality of the included studies, using the Cochrane Risk of Bias Assessment Tool to assess the risk of systematic errors in individual trials [21], incorporating six items: bias in the random allocation method, bias in allocation concealment, bias in blinding, bias in data integrity, bias with or without selective reporting, and other sources of risk of bias. A third member made the final decision if there was a discrepancy between two reviewers. This process was also applied to extraction of key variables. The quality of the literature was evaluated as high risk if one or more of the studies were assessed as high risk, low risk if there were no high-risk factors, and unknown risk if all were unclear.

### Data extraction

Two authors independently extracted the following pre-specified characteristics from all included randomized clinical trials: Subjects: age, sex, race, previous antiviral therapy, presence of cirrhosis, number of patients randomly assigned, baseline information at enrollment; Interventions: treatment dose, duration, method of administration; Observed efficacy indicators: HDV-RNA negative rate, ALT recurrence rate, and liver biopsy tissue improvement rate.

### Statistical analysis

This study is based on the framework of frequency science.Stata 17.0 software (Stata Corporation, College Station, TX, USA) was used to produce a mesh of direct and indirect comparisons between the outcomes of different treatment measures; the outcome indicators of this study were all dichotomous variables, and the results were expressed using relative risk (RR) and its 95% confidence interval (95% CI), with *P* < 0.05 indicating a significant difference. Pairwise meta-analyses meta-analysis was performed using Stata 17.0 software, and I^2^ was used to test for heterogeneity among studies; The node splitting method was used to check for inconsistency. In this study, SUCRA was used to calculate the cumulative ranking probability of each treatment scheme. The confidence in network meta-analysis (CINeMA) framework was applied to determine the certainty of evidence.

## Results

### Search results and basic characteristics of the literature

#### Search results

**Fig. 1:**
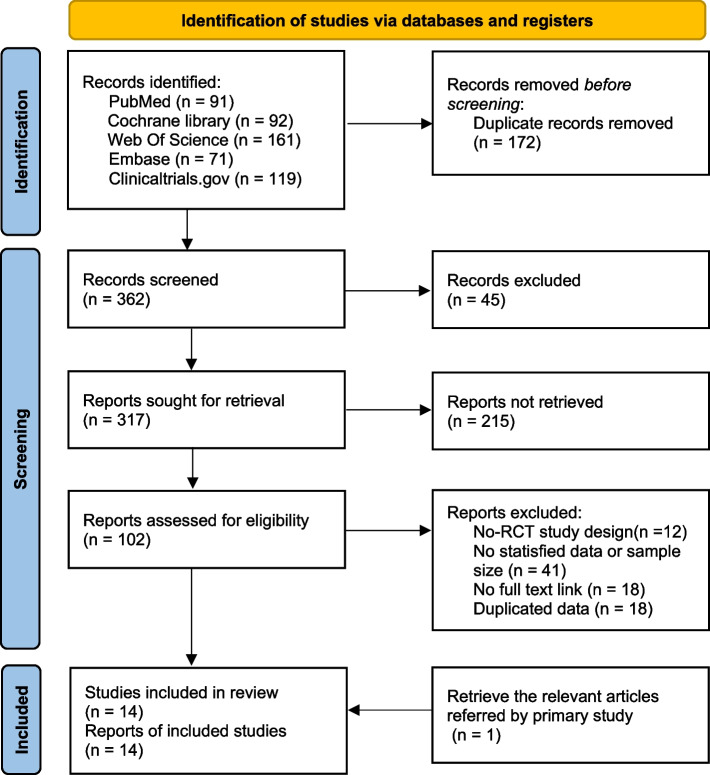
Flow chart of literature screening

A total of 520 studies were detected according to the search strategy and other routes, of which 14 clinical randomized controlled trials (RCTs) were included in the final network meta-analysis. All the studies were written in English, and a flow chart of the literature screening is shown in Fig. 1.

#### Basic characteristics of the included studies

The 14 included studies contained 814 patients with chronic hepatitis D, and all were included in the data analysis; 578 of patients were males and 236 were females. There were no significant differences in age, sex, disease duration, or severity of illness between the reporting groups, and the sample size was bounded by 20–120 patients. One study was a four-arm study [22] and three studies were three-arm studies [10, 23, 24]. The remaining 10 studies were two-arm studies [11, 25–33]. The study sites included Pakistan, Turkey, Italy, France, Germany, Greece, and Russia. A total of seven treatment regimens were reported, including BLV, IFN, Nucleoside analogs (NAs), BLV + IFN, BLV + NAs, IFN + NAs, and blank controls (or placebo), among which IFN was administered subcutaneously, NAs and BLV were administered orally, the shortest treatment was 24 weeks, the longest treatment was 96 weeks, and the longest follow-up was 365 weeks. The basic characteristics of the included studies are shown in Table 1.

**Table 1:** Basic characteristics of included studies

Study	Country	Treatment duration	Followed-up duration	Arms (No.)	Interventions	Sample sizes	Sex (M/F)	Age (Mean ± SD)
Abbas, Z (2016) [25]	Pakistan	72W	96W	2	IFN + NAs	21	16/5	26.4 ± 6.4
					IFN	19	15/4	27 ± 7.4
Canbakan, B (2006) [26]	Turkey	48W	95W	2	IFN	12	8/4	43.83 ± 8.57
					IFN + NAs	14	7/7	42.5 ± 11.02
Farci, P (1994) [27]	Italy	48W	96W	2	IFN	28	22/6	35 ± 8.4
					No treatment	14	13/1	37 ± 12
Gaudin, J. L (1995) [28]	France	48W	24W	2	IFN	11	11/0	29.6 ± 17.3
					No treatment	11	11/0	34.8 ± 39
Niro, G. A (2006) [29]	Italy	72W	96W	2	IFN	16	8/8	45.4 ± 8.8
					IFN + NAs	22	15/7	43 ± 9.6
Porres, J. C (1989) [30]	Spain	24W	60W	2	IFN	10	7/3	25.8 ± 9.6
					No treatment	10	8/2	31.2 ± 11.4
Rosina, F (1991) [31]	Italy	48W	96W	2	IFN	31	26/5	30 ± 2
					No treatment	30	28/2	29 ± 2
Rosina, F (1990) [32]	Italy	48W	96W	2	IFN	26	23/3	31.04 (19–41)^a^
					No treatment	22	21/1	29.16 (18–59)^a^
Wedemeyer, H (2011) [10]	Germany, Turkey, Greece	48W	72W	3	IFN + NAs	31	20/11	42 (23–59)^b^
					IFN	29	17/12	38 (17–62)^b^
					NAs	30	19/11	33 (21–55)^b^
Wedemeyer, H (2019) [11]	Germany, Greece, Romania, Turkey	96W	365W	2	IFN + NAs	59	38/21	38.1 ± 12.3
					IFN	61	41/20	42.1 ± 10.3
Wranke, A (2020) [33]	Germany, Greece, Turkey	48W	24W	3	IFN + NAs	19	NA	NA
					IFN	20	NA	NA
					NAs	21	NA	NA
Yurdaydin, C (2008) [23]	Turkey	48W	72W	3	NAs	17	15/2	38 (20–55)^b^
					IFN + NAs	14	10/4	35 (20–48)^b^
					IFN	8	8/0	46 (38–67)^b^
Hepatera Ltd. (2016) [22]	Russia	48W	72W	4	IFN	15	5/10	34.7 ± 7.1
					BLV + IFN	45	30/15	36.5 ± 6.7
					BLV	15	11/4	42 ± 9.6
					BLV + NAs	15	11/4	34.3 ± 7.2
Ltd, H. (2016) [24]	Russia, Germany	24W	48W	2	BLV + NAs	90	59/31	40.7 ± 9.7
					NAs	28	20/8	38.5 ± 8.7

*NA* Not available, *F* Female, *M* Male

^a^Mean ± SD

^b^Median (range)

#### Risk of bias

**Fig. 2:**
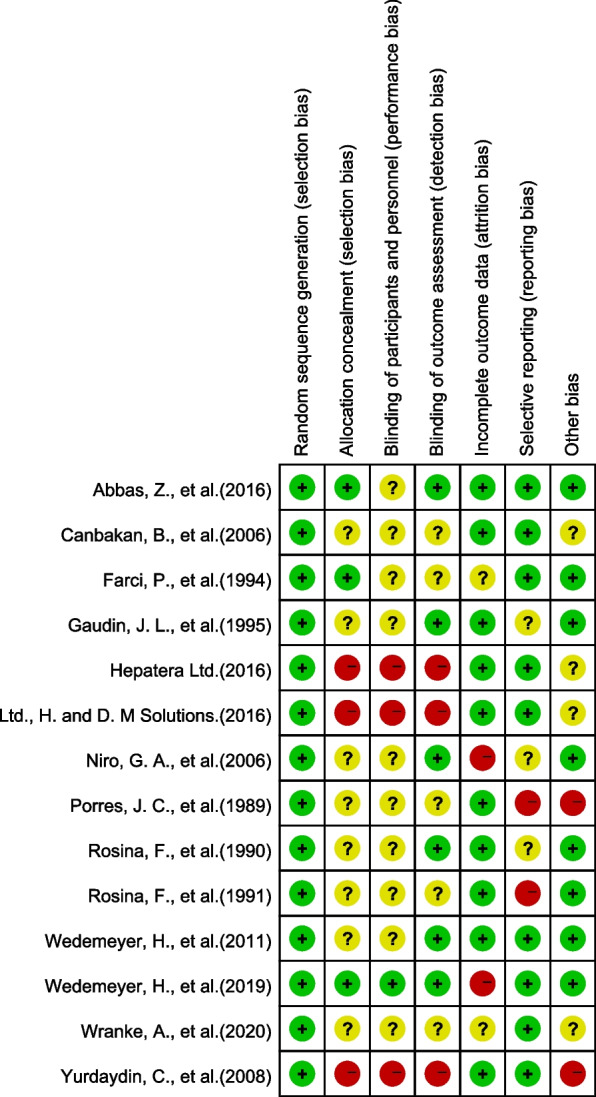
Risk of bias assessment

**Fig. 3:**
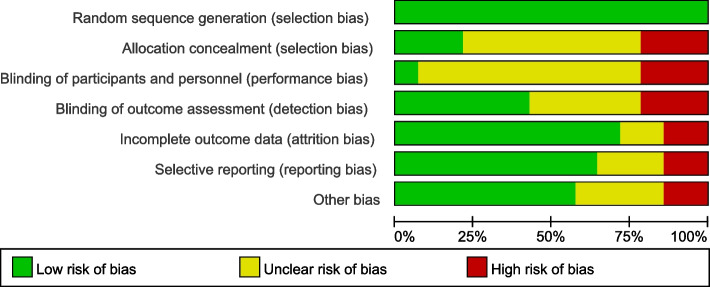
Summary of risk of bias assessment

The Cochrane Risk of Bias Assessment Tool was used to assess the risk of systematic error in individual trials [21]. Nearly half of the trials had a high risk of bias, with possible bias mainly in terms of allocation protocols not hidden and blinding not mentioned; the results are summarized in Figs. 2 and 3.

Random assignment: All included trials were RCTs, five of which were allocated according to computer-generated sequences. In the other nine studies, the authors did not give sufficient details about the methods used, except to say that the patients were randomly assigned to the two groups. Allocation scheme concealment: Two studies [25, 27] described studies using sealed envelope concealment, one study [11] used central allocation and trait-identical drugs for allocation concealment, three open label trials [22–24] did not conceal the allocation method, and other included trials did not mention the concealment scheme. Blinding: One study [11] mentioned double-blinding to investigators and reviewers; one study [25] mentioned single-blinding to outcome reviewers; four studies [10, 28–32] described blinding to pathologists; three studies [22–24] conducted open label trials; and the remaining studies did not mention blinding. Data completeness: Most of the included studies had complete data; in two studies [11, 27], the missing values were directly attributed to nonresponse. There were cases of loss to follow-up in two studies [27, 33]; however, the reasons for the loss were not mentioned, making it difficult to judge the completeness of the data. Nonetheless, we chose to assess outcomes according to established criteria for a sustained response after treatment, which provided a fair representation of the trials. Selective reporting: Two studies [30, 32] did not report the designated main outcome indicators in advance; three studies [28, 29, 31] did not indicate the scoring rules for liver fibrosis; and the remaining studies were determined to have no selection bias, reporting the intended outcome for each patient included according to the trial report. Other biases: Baseline comparability: except for two studies [22, 24] that did not describe component baseline differences in detail, the remaining studies reported similar baseline characteristics of patients between groups; analytical unit bias: as mentioned above, there was significant methodological heterogeneity between these trials. In four studies [10, 22, 23, 33], multiple treatment groups were used. These groups were redefined to ensure simplified pairwise comparisons for representative analyses, which might have resulted in potential analytical unit bias in the meta-analysis.

### The results of meta-analysis

#### Randomized controlled trial evidence

**Fig. 4:**
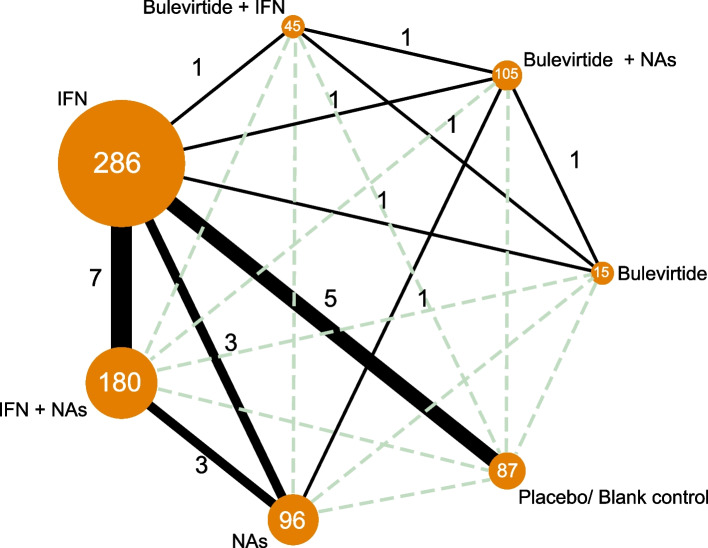
Network of randomized controlled trial evidence. The size of the nodes is proportional to the total number of participants allocated to each intervention and the thickness of the lines proportional to the number of studies evaluating each direct comparison

Seven treatment regimens for chronic hepatitis D could form 21 different pairwise comparisons. The 14 studies included in this network meta-analysis generated 10 direct comparisons and the remaining 11 had no evidence of direct comparisons, for which comparisons of efficacy outcomes were generated indirectly by the network analysis, as shown in Fig. 4.

#### Heterogeneity test and inconsistency test

**Fig. 5:**
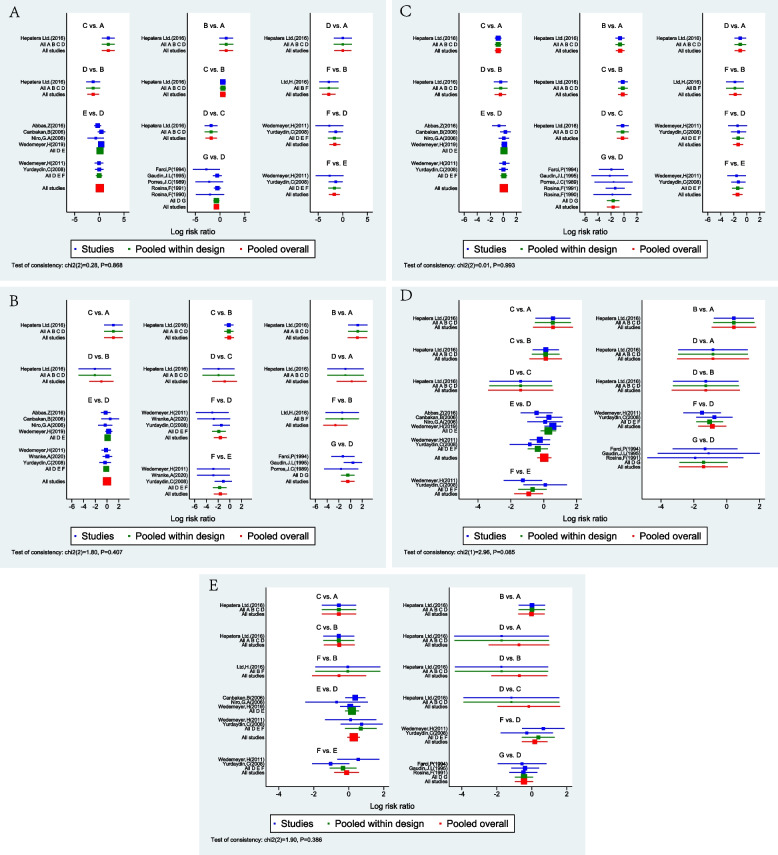
Consistency check. A: Bulevirtide; B: Bulevirtide + NAs; C: Bulevirtide + IFNα; D: IFNα; E: IFNα + NAs; F: NAs; G: No treatment; **A** Virological response (EOT); **B** Virological response (FU24W); **C** Biochemical response (EOT); **D** Biochemical response (FU24W);5E: Histological response

Direct meta-analysis was applied to analyze the heterogeneity among the studies, as shown in Table 2. The results showed that the *P* values were all > 0.05, suggesting no statistical heterogeneity among the clinical studies; therefore, the fixed effect model was used for analysis. A consistency test was also applied, and the *P* value was > 0.05 (Fig. 5). The node splitting method was used to check for inconsistency, and the results showed that the *P* value was > 0.05, indicating good consistency; therefore, the network meta-analysis was conducted under the consistency model.

**Table 2:** Direct comparison and homogeneity test among interventions

Intervertion			Virological response (EOF)				Virological response (FU24W)				Biochemical response (EOF)				Biochemical response (FU24W)				Histological response			
			RR	(95% CI)	HM		RR	(95% CI)	HM		RR	(95% CI)	HM		RR	(95% CI)	HM		RR	(95% CI)	HM	
					I^2^ (%)	*p*-value			I^2^ (%)	*p*-value			I^2^ (%)	*p*-value			I^2^ (%)	*p*-value			I^2^ (%)	*p*-value
A	vs	B	0.37	(0.09, 1.56)	-	-	0.27	(0.04, 2.07)	-	-	1.48	(0.66, 3.33)	-	-	0.71	(0.20, 2.53)	-	-	1.00	(0.38, 2.61)	-	-
A	vs	C	0.26	(0.07, 0.98)	-	-	0.30	(0.04, 2.12)	-	-	1.69	(0.90, 3.17)	-	-	0.65	(0.22, 1.95)	-	-	1.55	(0.48, 4.93)	-	-
A	vs	D	1.00	(0.16, 6.3)	-	-	2.20	(0.10, 49.06)	-	-	2.01	(0.76, 5.35)	-	-	2.06	(0.25, 17.34)	-	-	3.75	(0.25, 57.45)	-	-
B	vs	C	0.71	(0.37, 1.36)	-	-	1.11	(0.46, 2.71)	-	-	1.14	(0.51, 2.56)	-	-	0.91	(0.39, 2.14)	-	-	1.55	(0.56, 4.29)	-	-
B	vs	D	2.71	(0.64, 11.4)	-	-	6.05	(0.37, 99.49)	-	-	1.36	(0.45, 4.09)	-	-	2.90	(0.39, 21.7)	-	-	3.70	(0.25, 54.07)	-	-
B	vs	F	10.88	(1.57, 75.48)	-	-	3.89	(0.23, 66.95)	-	-	4.62	(1.18, 18.05)	-	-	-	-	-	-	1.04	(0.15, 7.10)	-	-
C	vs	D	3.84	(1.02, 14.41)	-	-	5.30	(0.34, 82.77)	-	-	1.19	(0.45, 3.15)	-	-	3.17	(0.47, 21.43)	-	-	2.50	(0.16, 39.05)	-	-
D	vs	E	0.93	(0.67, 1.28)	0.00	0.83	0.99	(0.70, 1.39)	0.00	0.96	0.95	(0.70, 1.29)	0.00	0.94	0.93	(0.67, 1.28)	0.00	0.57	0.88	(0.56, 1.37)	0.00	0.95
D	vs	F	7.28	(1.47, 35.99)	0.21	0.26	12.25	(2.39, 62.81)	0.33	0.22	2.99	(1.14, 7.88)	0.00	0.76	2.64	(1.14, 6.12)	0.00	0.42	1.05	(0.36, 3.07)	0.00	0.77
D	vs	G	2.55	(1.38, 4.73)	0.21	0.28	1.93	(0.61, 6.17)	0.00	0.38	6.09	(1.92, 19.33)	0.00	0.94	5.73	(0.90, 36.5)	0.00	0.75	1.39	(0.77, 2.52)	0.00	0.97
E	vs	F	6.11	(1.45, 25.7)	0.01	0.32	1.93	(0.61, 6.17)	0.00	0.38	3.14	(1.27, 7.8)	0.00	0.67	1.87	(0.79, 4.46)	0.35	0.22	1.65	(0.86, 3.16)	0.00	0.64

A: Bulevirtide, B: Bulevirtide + NAs, C: Bulevirtide + IFNα, D: IFNα, E: IFNα + NAs, F: NAs, G: No treatment

*HM* Heterogeneity measures

#### Network meta-analysis

##### Virological response

**Fig. 6:**
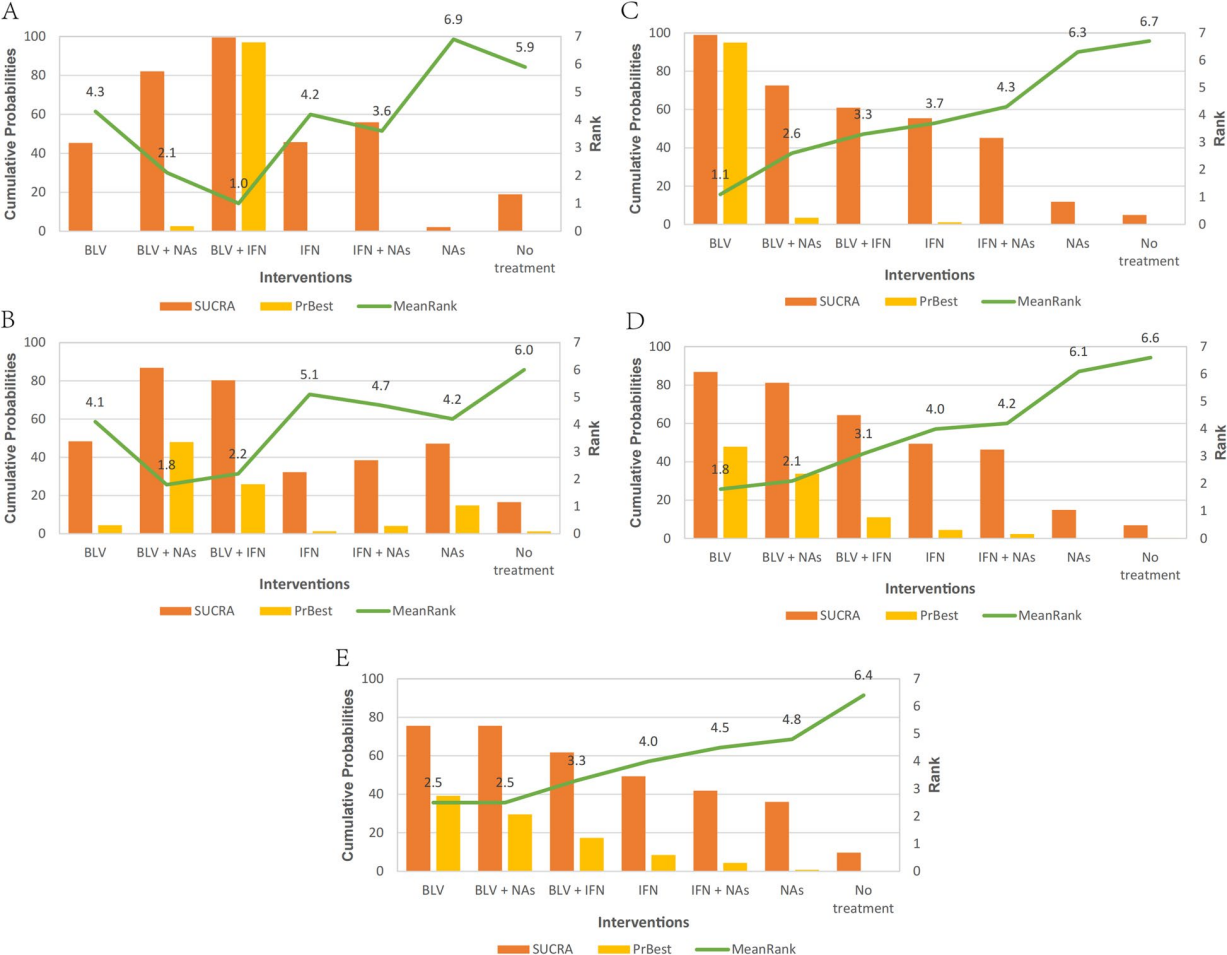
SUCRA probability ranking table. **A** Virological response (EOF); **B** Virological response (FU24W); **C** Biochemical response (EOF); **D** Biochemical response (FU24W); **E** Histological response

At the end of treatment, 754 patients from 13 trials were included. BLV + IFN, IFN monotherapy, IFN + NAs, and NAs monotherapy had better efficacy than the blank control group in HDV RNA clearance (RR = 17.24, 95% Cl [4.47,66.53], RR = 30.44, 95% Cl [7.86,117.81], RR = 4.99, 95% Cl [1.65,15.14], RR = 5.65, 95% Cl [1.87,17.10]). BLV + IFN and IFN monotherapy were more effective than other antiviral interventions (ORs ranging between 3.45 and 6.10), whereas BLV and BLV + NAs were among the least efficacious interventions (ORs ranging between 0.08 and 0.48). (Table 3A). Combining the results of direct and indirect comparisons, the HDV-RNA negative conversion rate (SUCRA) for each intervention was ranked from highest to lowest: BLV + IFN (99.5%) > BLV + NAs (82.1%) > IFN + NAs (56%) > IFN alone (45.8%) > BLV (45.4%) > blank control (19%) > NAs monotherapy (2.1%) (Fig. 6A). At 24 weeks of follow-up, 693 patients from 12 studies were included. BLV + IFN, IFN monotherapy, IFN + NAs, and NAs monotherapy were superior to the blank control (RR = 13.30, 95% Cl [1.68,105.32], RR = 12.13, 95% Cl [1.46, 101.04], RR = 5.05, 95% Cl [1.68,15.19], RR = 5.03,95% Cl [1.66,15.20]). There was no significant difference between the other interventions (Table 3B). Combining the results of direct and indirect comparisons, the interventions were ranked (SUCRA) from the highest to the lowest: BLV + NAs (86.8%) > BLV + IFN (80.3%) > BLV alone (48.4%) > NAs alone (47.1%) > IFN + NAs (38.5%) > IFN alone (32.3%) > Blank control (16.6%) (Fig. 6B).

**Table 3:**
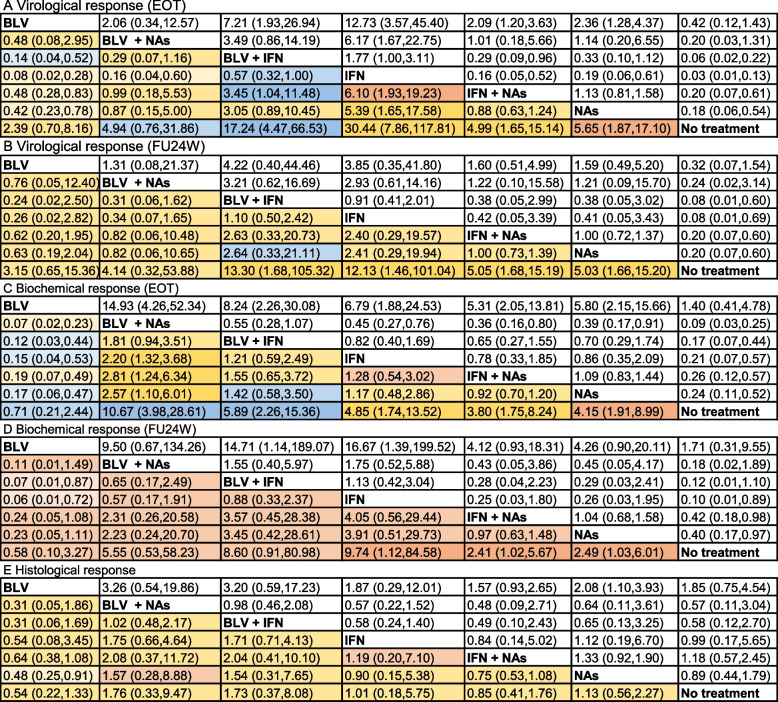
Indirect evidence from network meta-analysis with different interventions

Moderate, inferior  Low, inferior  Very low, inferior

Moderate, uncertain  Low, uncertain  Very low, uncertain

Moderate, superior  Low, superior  Very low, superior

##### Biochemical response

At the end of treatment, 754 patients from 13 trials were included. BLV + NAs, BLV + IFN, IFN monotherapy, IFN + NAs, and NAs monotherapy were superior to the blank control group in ALT normalization (RR = 10.67, 95% Cl [3.98,28.61], RR = 5.89, 95% Cl [2.26,15.36], RR = 4.85, 95% Cl [1.74,13.52], RR = 3.80, 95% Cl [1.75,8.24], RR = 4.15, 95% Cl [1.91,8.99]). Among them, BLV + NAs was more effective than other antiviral interventions (ORs ranging between 2.20 and 2.81) (Table 3C). Combining the results of direct and indirect comparisons, the probability ranking (SUCRA) of the ALT reversion rate for each intervention was, from the highest to the lowest: BLV alone (99%) > BLV + NAs (72.6%) > BLV + IFN (61%) > IFN + NAs (55.5%) > IFN alone (45.2%) > NAs alone (11.8%) > Blank control (4.9%) (Fig. 6C). At 24 weeks of follow-up, 556 patients from 10 studies were included. BLV + IFN and IFN monotherapy were superior to BLV monotherapy (RR = 14.71, 95% Cl [1.14,189.07], RR = 16.67, 95% Cl [1.39,199.52]), and IFN monotherapy, IFN + NAs were superior to the blank controls (RR = 9.74, 95% Cl [1.12,84.58], RR = 2.41, 95% Cl [1.02,5.67]), with no significant differences between the remaining interventions (Table 3D). Combining the results of direct and indirect comparisons, the probability ranking (SUCRA) of each intervention was, from the highest to the lowest: BLV + IFN (86.9%) > BLV + NAs (81.2%) > BLV alone (64.3%) > IFN + NAs (49.4%) > IFN alone (46.4%) > NAs alone (14.9%) > Blank control (6.9%) (Fig. 6D).

##### Histological response

Three hundred five patients from 10 studies were included. NAs alone were superior to BLV alone (RR = 2.08, 95% Cl [1.10, 3.93]) in terms of histological improvement on liver biopsy before and after treatment, with no significant differences between the remaining interventions (Table 3E). Combining the results of direct and indirect comparisons, the probability ranking (SUCRA) of the histological improvement of each intervention was, from the highest to the lowest: BLV monotherapy (75.6%) ≈ BLV + NAs (75.6%) > IFN + NAs (61.8%) > NAs monotherapy (49.4%) > BLV + IFN (41.9%) > IFN monotherapy (36%) > blank control (9.7%) (Fig. 6E).

#### Publication bias

**Fig. 7:**
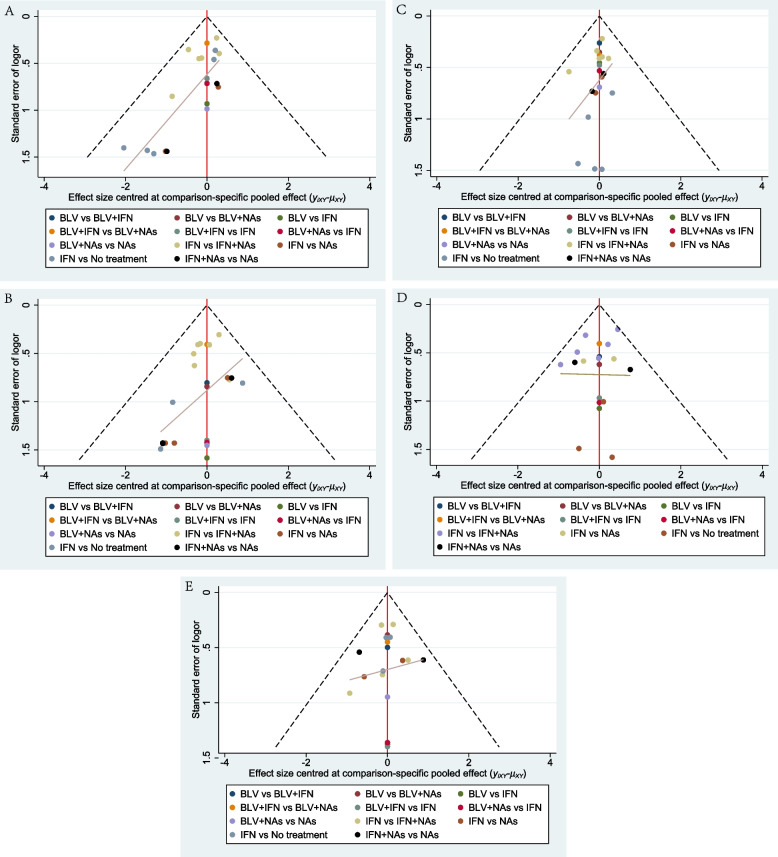
Bias detection comparison-correction funnel plot. **A** Virological response (EOT); **B** Virological response (FU24W); **C** Biochemical response (EOT); **D** Biochemical response (FU24W); **E** Histological response

There was no publication bias for the virological response, biochemical response at the end of treatment, and virological response at 24 weeks of follow-up; however, the funnel plot had poor symmetry for biochemical response at 24 weeks of follow-up and histological improvement before and after treatment, thus the study might have been subject to publication bias (Fig. 7).

#### Certainty of the evidence

For all outcomes, the certainty of evidence for most treatment comparisons was low (Table 3). For the outcomes, we judged the confidence of the evidence of 83% for comparison with control/blank group was low or very low. For the comparisons between BLV + IFN and IFN, the confidence of evidence of 80% was very low (Additional file 2).

## Discussion

The liver damage caused by HDV can induce severe or fulminant hepatitis, with severe clinical symptoms and liver dysfunction, resulting in a high case fatality rate. In this study, 14 randomized controlled trials involving 814 patients with chronic hepatitis D were included to assess the efficacy of different pharmacological intervention regimens in improving virological, biochemical, and histological response rates. The results show that: (1) IFN, IFN + BLV, and IFN + NAs have significant effects on the treatment of chronic hepatitis D, which can effectively clear HDV RNA and normalize ALT levels. However, more than half of the patients receiving IFN and IFN + NAs had HDV RNA positivity again at 24 weeks of follow-up after treatment. (2) Compared with IFN monotherapy, there was no additional benefit of adding NAs to IFN therapy for chronic hepatitis D, while IFN plus BLV significantly increased short-term HDV RNA clearance (36% *vs*. 82%, *p* < 0.05). (3) No significant benefit was shown for BLV monotherapy in the treatment of patients with chronic hepatitis D. BLV combination with IFNs and NAs had the second or third ranking in terms of virological, biochemical, and histological response in the short and medium term. It should be noted that only one study compared the efficacy of BLV alone with BLV + NAs and BLV + IFN, and it is necessary to conduct additional studies to further corroborate the comparative results. (4) No pharmacological intervention was effective in improving the inflammatory response or fibrosis of liver tissue.

Our results contrasted with those of previous studies of BLV. As a competitive inhibitor of hNTCP, BLV can effectively prevent HDV RNA entering liver cells and spreading, and even completely prevent the virus from entering at a very low concentration, as previously demonstrated using in vitro and in vivo models [15, 16, 34, 35]. The MYR202 study [17] is one of the largest trials to study BLV, and its results were included in this meta-analysis. This trial showed that up to 77% of patients receiving 10 mg of BLV achieved at least a 2-log or undetectable decline in HDV RNA; however, more than half of patients receiving BLV alone experienced virological relapse. A recent phase Ib/IIa study also demonstrated a strong effect of BLV on serum HDV RNA clearance, ALT normalization, and 5 of 7 patients who were treated with BLV in combination with interferon achieved sustained viral clearance. The combination of BLV and interferon showed strong antiviral synergy compared with monotherapy [36]. At present, the mechanism of co-inhibition of HDV by interferon and BLV is not completely clear. This is consistent with our study analysis: 87% of patients treated with bulevirtide + interferon achieved HDV RNA clearance at the end of treatment, which was significantly better than that of the other treatment regimens, and had strong effects of continuous HDV RNA clearance and ALT normalization. However, more clinical research data are needed to support the optimal dose and long-term effects of the combination regimen of BLV + IFN.

Consistent with previously published meta-analyses [8, 9], patients with chronic hepatitis D treated with IFN monotherapy showed some benefit in terms of viral clearance and ALT reversion, with 37% of them experiencing HDV RNA recurrence within 24 weeks after the end of treatment, although the addition of nucleoside analogs did not improve the viral response rate. However, the viral recurrence rate in the study was far less than that in the study by Abbas Z et al. [8], which might be related to the combination of IFN treatment with the PEG-IFN treatment group in that study. Some studies have shown that standard-dose PEG-IFN is more effective than high-dose IFN, with the sustained virological response rates of 29% [95% CI (19,41)] and 19% [95% CI (10,29)], respectively [37]. However, the effectiveness of PEG-IFN is also limited in patients with chronic hepatitis D. A meta-analysis of the effectiveness of PEG-IFN showed that viral clearance and normalization of ALT levels were achieved in only about one-third of patients with chronic hepatitis D at 24 weeks of follow-up after treatment [38]. Moreover, in the long-term follow-up of patients who had achieved a sustained virological response at 6 months post-treatment, delayed HDV RNA recurrence was found in more than half of the patients [39]. The superiority of PEGylated interferon needs to be further evaluated in more clinical trials.

Notably, while previous meta-analyses included only direct comparisons between IFNs, IFN + NAs, and placebo (or blank controls), this study has the advantage of comprehensively and simultaneously assessing the effectiveness of all drugs for the treatment of chronic hepatitis D, including the effectiveness of BLV, a hot spot in recent research. It provides data to support patients and physicians in choosing the optimal solution for the treatment of chronic hepatitis D.

It must be acknowledged that the results of this meta-analysis were limited by various factors. First, Despite the methodological rigor, this review has limitations related to the small number of randomized controlled trials included, resulting in only one study contributing to some comparison, with insufficient number of patients for allowing precise estimates. As each direct comparison arm had only one study, any bias in these studies could have affected the results of the network meta-analysis. Second, Most of the studies in this review were plagued by allocation, selective reporting, and data incompleteness bias. The evaluation was based on the intention-to-treat principle, and the possibility of publication bias cannot be excluded. The confidence of each comparison was assessed using the CINeMA method, and most comparisons were rated as “low” and “very low”. Therefore, further high-quality prospective randomized controlled trials are needed to compare the effectiveness of these treatment regimens. Third, although the results of this study suggest no observed benefit of these regimens in terms of liver histological improvement, the second biopsy in most studies was performed at the end of treatment rather than after a period of follow-up. Therefore, more data on the results of biopsies performed at follow-up after completion of treatment are needed for further evaluation. HBsAg is required for the production of viral hepatitis D particles. Ideally, the treatment of patients with chronic hepatitis D should continue until HBsAg loss. The impact of these treatment regimens on HBsAg clearance could not be explored because of the lack of sufficient data. Thus, the results of this meta-analysis should be interpreted with caution.

## Conclusion

In conclusion, based on the results of this meta-analysis, IFN, IFN + BLV, and IFN + NAs regimens were significantly effective in treating chronic hepatitis D, effectively clearing HDV RNA and normalizing ALT, but with a high rate of viral relapse with IFN and IFN + NAs at 24 weeks of post-treatment follow-up. Adding NAs to IFN therapy for chronic treatment provided no additional benefit, and the combination of IFN and BLV significantly improved short-term HDV RNA clearance, showing a strong synergistic effect. The seven regimens included in the study did not significantly contribute to liver histological improvement. Therefore, the combination of IFN and BLV has the potential to be the most effective treatment modality for chronic hepatitis D. Although the mechanism of action, dosing, and long-term efficacy associated with this therapy need to be further explored, it remains the most effective intervention available to reduce viral replication and disease activity. Many promising anti-HDV drugs are being developed, such as REP2139 and LNF. While waiting for new effective drugs, the application of BLV will likely improve the long-term prognosis of patients with chronic hepatitis D.

### Supplementary Information

**Additional file 1.** Search strategy.

**Additional file 2.**

## Data Availability

The datasets used and/or analysed during the current study are available from the corresponding author on reasonable request.

## Abbreviations

BLV: Bulevirtide
IFN: Interferon
NAs: Nucleoside analogs
RCTs: Randomized controlled trials
RR: Relative risk
Cl: Confidence interval
SUCRA: Surface under the cumulative ranking curve
HDV: Hepatitis D virus (or δ virus)
HBV: Hepatitis B virus
ALT: Alanine aminotransferase
LNF: Lonafarnib
HBsAg: HBV surface antigen
hNTCP: Human sodium taurocholate co-transporting polypeptide
EOT: End of treatment
FU24W: 24 Weeks of follow-up
