# Supplementary material for: Comparative effectiveness of seven interventions for chronic hepatitis D: a systematic review and network meta-analysis of randomized controlled trials
Source: BMC Infect Dis. 2023 Oct 25;23:726. doi: 10.1186/s12879-023-08718-7 (PMC10601284; doi:10.1186/s12879-023-08718-7)
Supplement: Supplementary file 1 — Additional file 1. Search strategy. [file 12879_2023_8718_MOESM1_ESM.docx]

**Additional file 1. Search strategy**

| **Embase** | **Search strategy** | **Results** |
| --- | --- | --- |
| **#1** | 'hepatitis d':ab,ti OR 'hepatitis d, chronic':ab,ti OR 'infection, delta':ab,ti OR 'hepatitis, delta':ab,ti OR 'delta hepatitis':ab,ti OR 'hepatitides, delta':ab,ti OR 'delta infection':ab,ti OR 'infections, delta':ab,ti OR 'superinfection, delta':ab,ti OR 'delta superinfections':ab,ti OR 'superinfections, delta':ab,ti OR 'delta superinfection':ab,ti OR 'chronic hepatitis d':ab,ti OR 'delta hepatitis, chronic':ab,ti OR 'chronic delta hepatitis':ab,ti OR 'chronic delta hepatitides':ab,ti OR 'delta hepatitides, chronic':ab,ti OR 'hepatitides, chronic delta':ab,ti | 4,503 |
| **#2** | **'randomized controlled trial':ab,ti OR 'randomized':ab,ti OR 'placebo':ab,ti OR 'rct':ab,ti** | **1070,562** |
| **#3** | **#1 AND #2** | **71** |

| **PubMed** | **Search strategy** | | **Results** | |  |
| --- | --- | --- | --- | --- | --- |
| **#1** | **"Hepatitis D"[Mesh] OR "Hepatitis D, Chronic"[Mesh]** | | **2,580** | |  |
| **#2** | **(Infection, Delta[Title/Abstract]) OR (Hepatitis, Delta[Title/Abstract]) OR (Delta Hepatitis[Title/Abstract]) OR (Hepatitides, Delta[Title/Abstract]) OR (Delta Infection[Title/Abstract]) OR (Infections, Delta[Title/Abstract]) OR (Superinfection, Delta[Title/Abstract]) OR (Delta Superinfections[Title/Abstract]) OR (Superinfections, Delta[Title/Abstract]) OR (Delta Superinfection[Title/Abstract]) OR (Chronic Hepatitis D[Title/Abstract]) OR (Delta Hepatitis, Chronic[Title/Abstract]) OR (Chronic Delta Hepatitis[Title/Abstract]) OR (Chronic Delta Hepatitides[Title/Abstract]) OR (Delta Hepatitides, Chronic[Title/Abstract]) OR (Hepatitides, Chronic Delta[Title/Abstract]) OR (Hepatitis, Chronic Delta[Title/Abstract])** | | **4,116** | |  |
| **#3** | **randomized controlled trial[Publication Type] OR randomized[Title/Abstract] OR placebo[Title/Abstract]** | | **977,506** | |  |
| **#4** | **#1 OR #2** | | **5,055** | |  |
| **#5** | **#3 AND #4** | | **91** | |  |
| **Cochrane library** | | **Search strategy** | | **Results** | |
| **#1** | | **MeSH descriptor: [Hepatitis, D] explode all trees** | | **58** | |
| **#2** | | **MeSH descriptor: [Hepatitis D,Chronic] explode all trees** | | **25** | |
| **#3** | | **("Hepatitis, D "):ab,ti,kw OR ("Hepatitis D,Chronic "):ab,ti,kw OR ("Infection, Delta"):ab,ti,kw OR ("Hepatitis, Delta"):ab,ti,kw OR ("Delta Hepatitis"):ab,ti,kw OR ("Hepatitides, Delta"):ab,ti,kw OR ("Delta Infection"):ab,ti,kw OR ("Infections, Delta"):ab,ti,kw OR ("Superinfection, Delta"):ab,ti,kw OR ("Delta Superinfections"):ab,ti,kw OR ("Superinfections, Delta"):ab,ti,kw OR ("Delta Superinfection"):ab,ti,kw OR ("Chronic Hepatitis D"):ab,ti,kw OR ("Delta Hepatitis, Chronic"):ab,ti,kw OR ("Chronic Delta Hepatitis"):ab,ti,kw OR ("Chronic Delta Hepatitides"):ab,ti,kw OR ("Delta Hepatitides, Chronic"):ab,ti,kw OR ("Hepatitides, Chronic Delta"):ab,ti,kw OR ("Hepatitis, Chronic Delta"):ab,ti,kw** | | **158** | |
| **#4** | | **(randomized controlled trial):ab,ti,kw OR (randomized):ab,ti,kw OR (placebo):ab,ti,kw OR (RCT):ab,ti,kw** | | **1153011** | |
| **#5** | | **#1 OR #2 OR #3** | | **158** | |
| **#6** | | **#5 AND #4** | | **92** | |

| **Web Of Science** | **Search strategy** | **Results** |
| --- | --- | --- |
| **#1** | **TS=("Hepatitis D" OR "Hepatitis D,Chronic" OR "Infection,Delta" OR "Hepatitis,Delta" OR "Delta Hepatitis" OR "Hepatitides,Delta" OR "Delta Infection" OR "Infections,Delta" OR "Superinfection,Delta" OR "Delta Superinfections" OR "Superinfections,Delta" OR "Delta Superinfection" OR "Chronic Hepatitis D" OR "Delta Hepatitis,Chronic" OR "Chronic Delta Hepatitis" OR "Chronic Delta Hepatitides" OR "Delta Hepatitides,Chronic" OR "Hepatitides,Chronic Delta" OR "Hepatitis,Chronic Delta")** and **Preprint Citation Index** (Exclude – Database) | **6,933** |
| **#2** | **TS=(randomized controlled trial OR randomized OR placebo OR RCT)** and **Preprint Citation Index** (Exclude – Database) | **1,288,697** |
| **#3** | **#1 AND #2** | **161** |
